# Assessment of Prescriber and Pharmacy Shopping Among the Family Members of Patients Prescribed Opioids

**DOI:** 10.1001/jamanetworkopen.2019.3673

**Published:** 2019-05-10

**Authors:** Kao-Ping Chua, Chad M. Brummett, Rena M. Conti, Rebecca L. Haffajee, Lisa A. Prosser, Amy S.B. Bohnert

**Affiliations:** 1 Department of Pediatrics, Susan B. Meister Child Health Evaluation and Research Center, University of Michigan, Ann Arbor; 2 Division of Pain Medicine, Department of Anesthesiology, University of Michigan Medical School, Ann Arbor; 3 Michigan Opioid Prescribing Engagement Network, Ann Arbor; 4 Institute for Health System Innovation and Policy, Department of Markets, Public Policy, and Law, Questrom School of Business, Boston University, Boston, Massachusetts; 5 Department of Health Management and Policy, University of Michigan School of Public Health, Ann Arbor; 6 Veterans Affairs Center for Clinical Management Research, Veterans Affairs Ann Arbor Health System, Ann Arbor, Michigan; 7 Department of Psychiatry, University of Michigan Medical School, Ann Arbor

## Abstract

**Importance:**

Most prescription opioid misuse involves opioids prescribed to others—a form of opioid diversion. However, few indicators of diversion risk exist. Because family members can often access patients’ opioids, one such indicator may be the frequency with which opioid prescriptions are filled by patients when their family members are engaged in opioid prescriber and pharmacy shopping (“doctor and pharmacy shopping”). To date, this frequency has not been estimated.

**Objective:**

To estimate the proportion of opioid prescription fills for which family members meet prescriber and pharmacy shopping criteria.

**Design, Setting, and Participants:**

A cross-sectional analysis of 2015-2016 claims from a national commercial insurer was conducted from August to October, 2018. The sample included patients without cancer who were covered by family insurance plans and had 1 or more opioid prescription fill in 2016, as measured by prescription drug claims. Fills were the unit of analysis.

**Main Outcomes and Measures:**

For each fill in 2016 by the patient and each family member enrolled in the same plan (eg, spouse or child), the number of prescribers and number of pharmacies in the prior 12 months were counted. Prescriber and pharmacy shopping was defined as 4 or more prescribers and 4 or more pharmacies, following a National Quality Forum–endorsed measure. The proportion of fills for which 1 or more family member met criteria and the proportion for which the patient met criteria were calculated.

**Results:**

Among 554 417 patients in the sample, 301 297 (54.3%) were female and 48 047 (8.7%) were children. Mean (SD) age was 41.4 (16.4) years. Patients were enrolled in 469 913 plans and, after exclusions, filled 1 471 971 opioid prescriptions in 2016. For 8485 fills (0.6%), 1 or more family member met prescriber and pharmacy shopping criteria. For 44 547 fills (3.0%), the patient met criteria. For 6947 of the 8485 fills (81.9%) for which 1 or more family member met criteria, patients did not meet criteria. When criteria were 3 or more prescribers at 3 or more pharmacies, the proportion of fills for which 1 or more family member met criteria increased to 1.9%.

**Conclusions and Relevance:**

In this national study of US patients with private family insurance plans, 0.6% of opioid prescription fills occurred when at least 1 of the patient’s family members met prescriber and pharmacy shopping criteria. For most of these fills, patients did not meet criteria. Findings suggest the potential for opioid diversion within families.

## Key Points

**Question:**

How frequently are opioid prescriptions filled by patients when their family members are engaged in prescriber and pharmacy shopping (“doctor and pharmacy shopping”)?

**Findings:**

In this cross-sectional analysis of nearly 1.5 million opioid prescription fills by 554 417 US adults and children with private family plans in 2016, 8485 (0.6%) fills occurred at the time that at least 1 family member met criteria for prescriber and pharmacy shopping.

**Meaning:**

Findings suggest the potential for opioid diversion within families and highlight the importance of avoiding excessive opioid prescribing.

## Teaser

This cross-sectional analysis examines prescriber and pharmacy shopping among patients who are prescribed opioids and their family members.

## Introduction

Prior studies indicate that the number of opioid prescribers and pharmacies used by patients are associated with opioid overdose death.^1,2,3,4,5,6,7,8,9,10,11^ In one study, the odds of opioid overdose death were 6.5 times higher when patients received opioid prescriptions from 4 or more vs fewer than 4 prescribers during the prior year, and 6.0 times higher when patients filled opioid prescriptions at 4 or more vs fewer than 4 pharmacies.^4^ Obtaining opioids from high numbers of prescribers and pharmacies, often referred to as prescriber and pharmacy shopping (“doctor and pharmacy shopping”), has been reported to be associated with greater risk of overdose death than high numbers of prescribers or pharmacies alone.^10,12^ To mitigate this risk, 49 states have implemented prescription drug monitoring programs (PDMPs), which allow clinicians and pharmacists to identify patients engaged in prescriber and pharmacy shopping by providing information on the prescribers and pharmacies of recent controlled substance prescriptions.^13,14^

The link between opioid overdose death and prescriber and pharmacy shopping is at least partially mediated by prescription opioid misuse, a behavior that includes using one’s own opioids in a manner other than prescribed.^15^ To facilitate efforts to prevent this type of misuse, the Pharmacy Quality Alliance recently developed a National Quality Forum–endorsed measure assessing use patterns consistent with prescriber and pharmacy shopping among patients without cancer who are prescribed opioids.^12^ However, prescription opioid misuse also includes taking someone else’s opioids, which are the source for misuse more than half of the time among the 4.2% of American individuals who misuse prescription pain relievers each year.^15^ Efforts by governments, payers, and clinicians to prevent this other type of misuse—a form of opioid diversion—would be facilitated by the development of risk indicators that are feasible to measure using existing data.

One indicator of diversion for the purpose of misuse may be the prevalence of prescriber and pharmacy shopping among the family members of patients prescribed opioids. Families are a natural unit of analysis because opioid use among patients and their family members can often be observed concurrently in data such as claims or electronic medical records. Furthermore, family members are among the most likely individuals to borrow, buy, or steal opioids belonging to patients, in large part because of their access to these opioids.^15,16,17^ To our knowledge, no study has estimated the frequency with which opioid prescriptions are filled by patients when their family members are engaged in prescriber and pharmacy shopping. The objective of this study was to estimate this frequency using a national commercial claims database and linkages between family members enrolled in the same health insurance plan.

## Methods

### Study Design and Data Source

From August to October 2018, we conducted a cross-sectional analysis of the 2015-2016 Clinformatics Data Mart (OptumInsight). This commercial claims database represents approximately 12 million patients each year who are covered by a large national insurer. Key data elements include unique family identifiers for enrollees in the same plan, as well as encrypted pharmacy identifiers and national provider identifier (NPI) data in prescription drug claims. Family plans cover the policyholder and dependents, such as spouses and children.

This study followed the Strengthening the Reporting of Observational Studies in Epidemiology (STROBE) reporting guideline.^18^ The institutional review board of the University of Michigan Medical School exempted this study from review because of its use of deidentified data.

### Opioid Prescription Fills

We defined an opioid prescription fill as a prescription drug claim for an opioid analgesic. We identified such claims using national drug codes compiled by the Centers for Disease Control and Prevention.^19^ We additionally included national drug codes identified by searching the *IBM Micromedex Red Book* for products including butorphanol, codeine, dihydrocodeine, fentanyl, hydrocodone, hydromorphone, levorphanol, meperidine, morphine, opium, oxycodone, oxymorphone, pentazocine, propoxyphene, tapentadol, or tramadol.^20^ Products containing methadone or buprenorphine were excluded, as these medications can be used both for analgesia and treatment of opioid use disorder.

### Sample

The sample included patients who (1) had 1 or more opioid prescription fills between January 1 and December 31, 2016, (2) were 2 years or older on December 31, 2016, (3) had continuous family insurance coverage throughout 2015 and 2016, and (4) had no *International Classification of Diseases, Ninth Revision–Clinical Modification* or *International Statistical Classification of Diseases, 10th Revision–Clinical Modification* diagnosis code for cancer on any claim during 2015 or 2016. We excluded patients with cancer to be consistent with the National Quality Forum–endorsed measure developed by the Pharmacy Quality Alliance (Use of Opioids From Multiple Prescribers in Persons Without Cancer).^12^

We used opioid prescription fills as the unit of analysis to capture the number of occasions in which information on family members’ opioid use patterns could inform prescribing decisions. We initially included all fills by patients in the sample during 2016 (index fills; the date was the index date). We excluded index fills if there was missing NPI data for any opioid prescription fill by the patient or any family members during a 12-month look-back period. This period began 366 days before the index date and ended 1 day before the index date.

### Study Variables

We defined prescriber and pharmacy shopping as filling opioid prescriptions from 4 or more prescribers at 4 or more pharmacies during the 12-month look-back period, following the specification of the Pharmacy Quality Alliance measure.^12^ This measure’s specification was informed by, but does not exactly follow, the criteria used in prior studies demonstrating associations between the risk of opioid overdose death and the number of prescribers and pharmacies.^1,2,3,4,5,6,7,8,9,10^ Measure validation occurred by assessing face validity among experts.^12^ To assess how results changed when using different criteria, we performed sensitivity analyses using more and less stringent criteria (≥4 prescribers and ≥4 pharmacies in 6 months, ≥3 prescribers and ≥3 pharmacies in 12 months, ≥4 prescribers in 12 months, and ≥4 pharmacies in 12 months).

### Statistical Analysis

For categorical variables, we calculated counts and frequencies. For continuous variables, we calculated means with SDs or medians with interquartile ranges (IQRs) when the distribution was skewed. Because of the study’s large sample size, the width of 95% CIs was narrow (eg, typically less than a few hundredths of a percentage point for proportions); consequently, we do not report these intervals.

We calculated the proportion of index fills for which 1 or more of the patient’s family members met criteria for prescriber and pharmacy shopping (primary outcome). We then independently repeated this analysis for the patient (secondary outcome). Family members were linked to patients using the unique family identifier and were eligible to meet prescriber and pharmacy shopping criteria only if they were 2 years or older at the end of 2016, had no cancer diagnosis in 2015 or 2016, and were continuously insured throughout 2015 and 2016. As an example, for index fills occurring on January 1, 2016, we identified prescription fills by each eligible family member that occurred between January 1, 2015, and December 31, 2015, and determined whether these fills were associated with 4 or more unique NPIs and 4 or more unique pharmacy identifiers. We then repeated this analysis for the patient.

Among index fills for which 1 or more family member met criteria, we calculated the proportion for which the patient did and did not also meet criteria. The latter represented instances in which PDMP reports would lack evidence of prescriber and pharmacy shopping by patients, even though their family members engage in this behavior.

Among index fills for which 1 or more family member met criteria, we determined the type and specialty of prescribing clinicians using taxonomy codes. Among index fills for which 1 or more family member did and did not meet criteria, we calculated median total morphine milligram equivalents (MMEs), a standardized measure of opioid amount.^19^ To assess whether the former group of index fills typically had a higher amount of opioid potentially available for diversion than the latter group, we assessed the significance of differences in total MMEs using a nonparametric test (Wilcoxon signed rank test), as the distribution of total MMEs was right skewed. We repeated this analysis for index fills for which the patient did and did not meet criteria.

To construct patient-level prevalence estimates, we calculated the proportion of patients who had 1 or more index fill in 2016 for which 1 or more family member met criteria, and the proportion who had 1 or more index fill in 2016 for which the patient met criteria. We also calculated the characteristics of family members who met criteria during 2016.

We conducted analyses both in the overall sample and by subgroups defined by age (index fills by adults ≥18 years vs children 2-17 years). We considered *P* values <.05 to be statistically significant and used 2-sided tests. Analyses were conducted using SAS, version 9.4 (SAS Institute Inc).

### Sensitivity Analysis

In addition to using different criteria for prescriber and pharmacy shopping, we performed a sensitivity analysis in which patients and family members with cancer were not excluded. We also repeated analyses that included buprenorphine and methadone as index fills.

## Results

### Sample

The database included 4 644 898 patients 2 years or older who had family insurance coverage throughout 2015 and 2016 and had no cancer diagnoses during this period. Among these patients, 554 417 individuals (11.9%) had 1 or more opioid prescription fill during 2016 and were included in the sample. Among patients in the sample, 301 297 (54.3%) were female, 506 370 (91.3%) were adults, and 48 047 (8.7%) were children (Table 1). Mean (SD) age was 41.4 (16.4) years. Patients in the sample represented 469 913 family plans. The mean (SD) number of family members per plan was 3.3 (1.3), the mean number of adults per plan was 2.5 (0.9), and the mean number of children per plan was 0.8 (1.1); 224 742 of the plans (47.8%) had at least one 1 adult member and at least 1 one child member.

**Table 1.** Demographic Characteristics of Study Sample

Characteristic	No. (%)
Total patients, No.	554 417
Female	301 297 (54.3)
Age, y	
2-11	13 485 (2.4)
12-17	34 562 (6.2)
18-25	80 467 (14.5)
26-34	54 717 (9.9)
35-44	107 138 (19.3)
45-54	127 094 (22.9)
55-64	109 935 (19.8)
≥65	27 019 (4.9)
Race/ethnicity	
White	400 870 (72.3)
Asian	17 805 (3.2)
Black	45 432 (8.2)
Hispanic	58 313 (10.5)
Unknown	31 997 (5.8)
Census region	
Northeast	39 209 (7.1)
Midwest	148 805 (26.8)
South	248 599 (44.8)
West	116 089 (20.9)
Unknown	1715 (0.3)

Patients in the sample filled 1 529 476 opioid prescriptions in 2016, of which 57 505 fills (3.8%) were excluded owing to missing NPI data for prescription fills by the patient or any family members during the look-back period, leaving 1 471 971 index fills. Of these, 1 415 483 fills (96.2%) were by adults and 56 488 fills (3.8%) were by children. The most common opioids were hydrocodone (613 276 [41.7%]), oxycodone (358 421 [24.4%]), tramadol (264 625) [18.0%]), and codeine (124 706 [8.5%]).

### Main Results

For 8485 of the 1 471 971 (0.6%) index fills, 1 or more family member met prescriber and pharmacy shopping criteria. For 44 547 of the index fills (3.0%), the patient met criteria (Table 2). For 1538 (0.1%) of the index fills, both the patient and 1 or more family member met criteria; these fills are reflected in both the 0.6% and 3.0% estimates above. Among 8485 index fills for which 1 or more family member met criteria, the patient did not meet criteria for 6947 (81.9%) of the fills, but did meet criteria for 1538 fills (18.1%).

**Table 2.** Prevalence of Prescriber and Pharmacy Shopping Among Opioid Prescription Fills

Prevalence Estimate	Overall (n = 1 471 971 Fills)	Adults Aged ≥18 y (n = 1 415 483 Fills)	Children Aged 2-17 y (n = 56 488 Fills)
No. of index fills in 2016 for which ≥1 family member met criteria (% of all fills)	8485 (0.6)	8102 (0.6)	383 (0.7)
No. of index fills in 2016 for which the patient met criteria (% of all fills)	44 547 (3.0)	44 416 (3.1)	131 (0.2)

Among 8485 index fills for which 1 or more family member met prescriber and pharmacy shopping criteria, 5782 (68.1%) of prescribing clinicians were physicians, 667 (7.9%) were physician assistants, 610 (7.2%) were nurse practitioners, and 363 (4.3%) were dentists. The most common physician specialties among the 8485 index fills were family medicine (1506 [17.7%]), pain medicine (1390 [16.4%]), internal medicine (812 [9.6%]), and orthopedic surgery (548 [6.5%]) (Table 3).

**Table 3.** Prescribers of Opioid Prescription Fills for Which 1 or More Family Member or the Patient Met Prescriber and Pharmacy Shopping Criteria

Prescriber Type	No. (% of All Fills)	
	≥1 Family Member Met Prescriber and Pharmacy Shopping Criteria (n = 8485)	Patient Met Prescriber and Pharmacy Shopping Criteria (n = 44 547)
Dentist	363 (4.3)	1015 (2.3)
Nurse practitioner	610 (7.2)	3616 (8.1)
Physician assistant	667 (7.9)	4022 (9.0)
Podiatrist	65 (0.8)	432 (1.0)
Miscellaneous^a^	257 (3.0)	1594 (3.6)
Unknown^b^	741 (8.7)	3944 (8.9)
Physician	5782 (68.1)	29 994 (67.3)
Family medicine	1506 (17.7)	6808 (15.3)
Internal medicine	812 (9.6)	4436 (10.0)
Pain medicine^c^	1390 (16.4)	7298 (16.4)
Orthopedic surgery	548 (6.5)	3518 (7.9)
Other surgical specialty	347 (4.1)	1522 (3.4)
Emergency medicine	430 (5.1)	1892 (4.2)
Physical medicine and rehabilitation^d^	284 (3.3)	1543 (3.5)
Obstetrics and gynecology	96 (1.1)	741 (1.7)
Psychiatry and neurology^d^	44 (0.5)	746 (1.7)
Anesthesiology^d^	134 (1.6)	807 (1.8)
Other physician specialty	191 (2.3)	633 (1.4)

^a^ Includes midwives, chiropractors, optometrists, and other midlevel clinicians.

^b^ Includes clinicians with missing provider taxonomy codes or codes corresponding to unknown.

^c^ Includes pain medicine specialists from anesthesiology, psychiatry, neurology, and physical medicine and rehabilitation, and physicians with taxonomy codes corresponding to “allopathic and osteopathic physicians–pain medicine and interventional pain medicine.”

^d^ Excludes physicians in these specialties who are also listed as pain medicine specialists.

Among index fills for which 1 or more family member did and did not meet prescriber and pharmacy shopping criteria, median (IQR) total MMEs were 450 (150-1200) and 300 (150-900), respectively (*P* < .001). Among index fills for which the patient did and did not meet criteria, median (IQR) total MMEs were 600 (225-1350) and 300 (150-900), respectively (*P* < .001).

### Patient-Level Prevalence Estimates

Among the 554 417 patients in the sample, 2455 individuals (0.4%) filled 1 or more opioid prescription in 2016 for which 1 or more family member met criteria for prescriber and pharmacy shopping. Of these 2455 patients, 2242 (91.3%) were adults, 213 (8.7%) were children, and 1043 (46.5%) were female. Among 2363 family members who met criteria at any point during 2016, 2339 (99.0%) were adults and 1472 (62.3%) were female. Among the 554 417 patients in the sample, 6582 individuals (1.2%) filled 1 or more opioid prescription for which they met criteria themselves.

### Subgroup Analysis by Age

Among index fills by adults, the proportion for which the 1 415 483 adults met prescriber and pharmacy shopping criteria (44 416 [3.1%]) was higher than the proportion for which 1 or more family member met criteria (8102 [0.6%]). In contrast, among index fills by 56 488 children, the proportion for which 1 or more family member met criteria (383 [0.7%]) was higher than the proportion for which the child met criteria (131 [0.2%]) (Table 2).

Among the 506 370 adult patients, 2242 individuals (0.4%) filled 1 or more opioid prescription in 2016 for which 1 or more family member met criteria, while 6564 adults (1.3%) filled 1 or more opioid prescription in 2016 for which the patient met criteria. Among the 48 047 child patients, 213 individuals (0.4%) filled 1 or more opioid prescription in 2016 for which 1 or more family member met criteria, and 18 children (0.04%) filled 1 or more opioid prescription in 2016 for which the patient met criteria. Among children with fills for which 1 or more family member met criteria, the mean (SD) age of these family members was 40.1 (7.6) years.

### Sensitivity Analyses

When prescriber and pharmacy shopping criteria were defined as 4 or more pharmacies and 4 or more pharmacies over a 6-month look-back period, the proportion of fills for which 1 or more family member met criteria decreased from 0.6% (n = 8485) to 0.2% (n = 2394). When criteria were defined as 3 or more prescribers and 3 or more pharmacies over 12 months, this proportion increased to 1.9% (n = 27 578). When requiring 4 or more prescribers over 12 months (prescriber shopping alone), this proportion was 2.6% (n = 38 543), compared with 1.4% (n = 20 003) when requiring 4 or more prescribers over 12 months (pharmacy shopping alone). Findings were virtually identical when including patients and family members with cancer, as well as when including buprenorphine and methadone as index fills (Table 4).

**Table 4.** Sensitivity Analyses

Analysis	Criteria	Look-Back Period	No. (% of All Fills)	
			Index Fills for Which ≥1 Family Member Met Criteria	Index Fills for Which Patient Met Criteria
Main analysis (n = 1 471 971 fills)	≥4 Prescribers and ≥4 pharmacies	12 mo	8485 (0.6)	44 547 (3.0)
Shorter 6-mo look-back period (n = 1 486 314 fills)^a^	≥4 Prescribers and ≥4 pharmacies	6 mo	2394 (0.2)	13 687 (0.9)
Fewer prescribers and pharmacies (n = 1 471 971 fills)	≥3 Prescribers and ≥3 pharmacies	12 mo	27 578 (1.9)	12 315 (8.2)
Prescriber shopping only (n = 1 471 971 fills)	≥4 Prescribers	12 mo	38 543 (2.6)	178 417 (12.1)
Pharmacy shopping only (n = 1 471 971 fills)	≥4 Pharmacies	12 mo	20 003 (1.4)	93 267 (6.3)
Include methadone and buprenorphine (n = 1 522 822 fills)	≥4 Prescribers and ≥4 pharmacies	12 mo	9184 (0.6)	47 661 (3.1)
Include patients and family members with cancer (n = 1 752 908 fills)	≥4 Prescribers and ≥4 pharmacies	12 mo	11 136 (0.6)	55 794 (3.2)

^a^ Number of index fills is higher than the main analysis because index fills were excluded if there was missing national provider identifier information on any opioid prescription fills by the patient or family members in the 6 months before the index date instead of 12 months, resulting in fewer exclusions.

## Discussion

In this national study of 554 417 patients who were enrolled in private family insurance plans and filled opioid prescriptions during 2016, 0.6% of the fills (1 of 167) occurred when at least 1 of the patient’s family members met criteria for opioid prescriber and pharmacy shopping. For most of these fills, patients did not meet criteria. Family members who are engaged in prescriber and pharmacy shopping may have both the motive and opportunity to divert patients’ opioids. Consequently, findings suggest the potential for opioid diversion within families.

To our knowledge, this study is the first to assess prescriber and pharmacy shopping among the family members of patients prescribed opioids. If the study’s prevalence estimate of 0.6% is nationally representative, then family members met prescriber and pharmacy shopping criteria for approximately 1.2 million opioid prescriptions in 2016, a year in which 210 million opioid prescriptions were filled in the United States.^21^ However, this estimate likely represents a lower bound. First, the study’s database did not capture opioid prescriptions paid for by cash, which is a common source of payment among patients engaged in opioid prescriber and pharmacy shopping.^1^ Second, the study only included commercially insured patients with family coverage for 24 months. Commercially insured patients have lower rates of prescriber and pharmacy shopping than other populations,^12^ while the ability to maintain family coverage for a prolonged period may be a marker of unobserved factors that decrease the likelihood of this behavior (eg, economic stability). Finally, the study used a somewhat conservative definition of prescriber and pharmacy shopping to be consistent with a national quality measure and avoid misclassification of legitimate use patterns.^8,12^ Based on these considerations, the absolute number of opioid prescriptions associated with prescriber and pharmacy shopping by family members is likely to be substantial. Prescriber and pharmacy shopping indicates a fairly extreme example of opioid misuse-related behavior. Less-extreme misuse-related behavior likely occurs in the family members of patients prescribed opioids, suggesting that this study’s findings may also underestimate the potential for opioid diversion within families.

In this study, 0.4% of patients had at least 1 opioid prescription fill in 2016 for which family members met prescriber and pharmacy shopping criteria, even though family members met these criteria for 0.6% of opioid prescription fills. This finding indicates that some patients had multiple such fills in 2016, suggesting that there were multiple opportunities to detect prescriber and pharmacy shopping among their family members. However, such detection may be difficult for clinicians. For 81.9% of opioid prescription fills for which family members met prescriber and pharmacy shopping criteria in this study, the patient did not meet criteria, implying that his or her PDMP reports would not contain evidence of this behavior. Therefore, the absence of this behavior on patients’ PDMP reports does not reliably indicate the absence of this behavior among their family members. Furthermore, findings suggest that prescriber and pharmacy shopping occurs in the family members of patients with both acute and chronic pain. Among opioid prescription fills for which family members met criteria in this study, prescribers included clinicians who often prescribe opioids for chronic pain (eg, family medicine and pain medicine physicians), as well as clinicians who often prescribe opioids for acute pain (eg, surgeons and dentists).

The amount of opioids potentially available for diversion was typically higher when fills were associated with prescriber and pharmacy shopping by family members than when they were not. One explanation is that the former were more likely to represent high-dose opioid prescriptions for chronic pain, in which case counseling on safe opioid storage and disposal may be an important intervention to prevent diversion.^22,23^ An alternative explanation is that family members engaged in prescriber and pharmacy shopping might sometimes accompany patients to visits and manipulate clinicians by suggesting a higher-than-needed opioid requirement. Yet another explanation is that some patients may attempt to obtain high-dose opioid prescriptions on behalf of family members engaged in prescriber and pharmacy shopping. In either of these latter 2 situations, counseling on safe storage and disposal will likely not prevent diversion. A more effective intervention may be to rely on nonopioid therapy when feasible and avoid opioid prescriptions with amounts that exceed clinical need, thus decreasing the amount of opioids available for diversion regardless of family dynamics.

Among opioid prescription fills by children in this study, family members met prescriber and pharmacy shopping criteria for 0.7%. Although the study’s database did not contain information on family relationships, the mean age of these family members was 40.1 years, suggesting that many may have been parents. Furthermore, 0.2% of fills by children met prescriber and pharmacy shopping criteria. Children lack the ability to obtain opioid prescriptions from multiple clinicians and fill them at multiple pharmacies by themselves, suggesting that apparent prescriber and pharmacy shopping behavior in children is likely driven by an adult family member. Collectively, these findings potentially support concerns among some pediatric clinicians that parents and other family members may misuse opioids prescribed to children or may feign or exploit pain in children to obtain opioids for themselves.^24^ Our analysis of PDMP statutes in October 2018 showed that 39 states mandate health care professionals to review PDMP reports under some circumstances before prescribing controlled substances to patients, including children.^13^ However, for opioid prescriptions to children in this study, family members were 3 times more likely than the child to meet prescriber and pharmacy shopping criteria. Thus, for children prescribed opioids, interventions focused on preventing misuse among family members may be more useful than interventions focused on children.

The challenges and potential importance of detecting prescriber and pharmacy shopping among family members raises the question of whether policy makers should increase clinicians’ access to information on family members’ opioid use patterns. In theory, for example, clinicians could be explicitly allowed to check family members’ PDMP reports if amendments were made to the Health Insurance Portability and Accountability Act,^25^ state privacy laws, and state PDMP laws that impose criminal penalties for wrongful use of PDMP data, defined typically as access under circumstances that do not pertain to a patient’s care or fall within an established clinician-patient relationship.^26^ However, such amendments are hard to justify given the relative rareness of prescriber and pharmacy shopping in family members and the importance of maintaining patient privacy to avoid stigma and other negative consequences. Consequently, interventions at the clinical level, such as avoiding excessive opioid prescribing, are likely the most feasible and appropriate method to prevent opioid diversion within families.

### Limitations

This study has several limitations. First, prescriber and pharmacy shopping was defined in part based on the number of unique NPIs associated with opioid prescription fills, but some opioid prescribers may have been assigned multiple NPIs. Second, the database did not capture opioid use patterns by family members who were not enrolled in the same insurance plan. Third, this study did not examine the consequences of prescribing opioids to patients whose family members are engaged in prescriber and pharmacy shopping, such as increased overdoses in these family members.

## Conclusions

Given the volume of opioid prescribing in the United States, the absolute number of opioid prescriptions associated with prescriber and pharmacy shopping by family members is likely to be substantial. Assessing the prevalence of this behavior using data such as claims may help policy makers understand and monitor the risk of opioid diversion within families. Clinicians should be aware of this potential and avoid opioid prescribing that exceeds clinical need.
